# Long-Term Survival and Medical Costs of Patients with Prolonged Mechanical Ventilation and Tracheostomy: A Nationwide Cohort Study

**DOI:** 10.3390/ijerph181910272

**Published:** 2021-09-29

**Authors:** Hui-Hsuan Lai, Pei-Ying Tseng, Chen-Yu Wang, Jong-Yi Wang

**Affiliations:** 1 Department of Nursing, China Medical University Hospital, Taichung 404332, Taiwan; u104090019@cmu.edu.tw; 2 Department of Public Health, China Medical University, Taichung 406040, Taiwan; cynthia522022@gmail.com; 3 Department of Medical, Lee’s General Hospital, Yuanli Town, Miaoli 358011, Taiwan; 4 Department of Critical Care Medicine, Taichung Veterans General Hospital, Taichung 407752, Taiwan; chestmen@gmail.com; 5 Department of Nursing, Hungkuang University, Taichung 433304, Taiwan; 6 Department of Health Services Administration, China Medical University, Taichung 406040, Taiwan

**Keywords:** tracheotomy, mechanical ventilator, mortality, survival analysis, medical expense

## Abstract

Few large-scale studies have focused on tracheostomy in patients with prolonged mechanical ventilation. This retrospective population-based study extracted data from the longitudinal National Health Insurance Research Database in Taiwan to compare long-term mortality between patients on prolonged mechanical ventilation with and without tracheostomy and their related medical expenditures. Data on newly developed respiratory failure in patients on ventilator support were extracted from 1 January 2002 to 31 December 2008. Of 10,705 patients included, 1372 underwent tracheostomy (n = 563) or translaryngeal intubation (n = 779). Overall survival of the patients with tracheostomy was followed for 5 years. Average survival was 4.98 years for the patients with tracheostomy and 5.48 years for the patients with translaryngeal intubation (not significant). Sex, age, premium-based monthly salary difference, occupation, urbanization level, chronic obstructive pulmonary disease, chronic heart failure, chronic renal disease, and cerebrovascular diseases were significantly associated with mortality for endotracheal intubation. Male sex, chronic heart failure, chronic renal disease, age ≥45 years, and low income were associated with significantly higher mortality. Although total medical expenditures were higher for the patients with tracheostomy, annual medical expenditures were not significantly different. There were no differences in long-term mortality between the two groups.

## 1. Introduction

Prolonged translaryngeal endotracheal intubation increases the risk of laryngeal injury, sinusitis, and ventilator-associated pneumonia [1,2,3]. Hence, tracheostomy is preferred to translaryngeal endotracheal intubation, because it provides better oral hygiene, improved patient comfort (Table 1), returned speech, and airway security during weaning [4,5,6]. However, the optimal timing for tracheostomy remains a clinical dilemma, with inconclusive results in the literature due to the heterogeneity of critically ill patients [7].

Prolonged mechanical ventilation (PMV) is defined as a period of ≥6 h/day on mechanical ventilation for 21 days, and it has been suggested that early tracheostomy after 2 weeks of ventilator dependency could be considered, based on patient comfort [7,8,9,10]. A retrospective study showed that the weaning rate from a ventilator was 56.3% [11], with old age [12], chronic obstructive pulmonary disease (COPD) [13], poor heart function [14], high APACHE II score [15], and lower Glasgow Coma Scale score [16] causing difficulty in weaning. Thus, physicians and family inevitably face a difficult decision regarding the timing of tracheostomy for patients with PMV.

Reportedly, early tracheostomy does not decrease hospital [17] or 30-day mortality [18]. Moreover, there were no differences in 1-year mortality between early tracheostomy, late tracheostomy, and translaryngeal endotracheal intubation [19]. In a cohort study in Germany, the survival rate of ventilated patients discharged from hospital was 67.6% within 3 months, 49.4% within 1 year, and 38.1% within 3 years [13]. Pilcher et al. also showed that the 3-year survival rate of patients with tracheostomy who were successfully weaned from the ventilator was 47% [20]. In the US, the number of ventilated patients who received a tracheostomy increased from 16.7 to 34.3 per 100,000 adults from 1993 to 2002, and only 10% of the patients were successfully discharged to home, with the need for long-term care after discharge increasing from 40.1% to 71.9% [21]. Long-term care of patients with PMV is associated with increased medical expenditures. Although the long-term outcomes of patients with PMV have been discussed in the literature, few large-scale studies have focused on patients with PMV and tracheostomies. Indeed, some patients and their families remain hesitant to undergo tracheostomy due to insufficient information regarding the long-term outcome of patients with tracheostomy. The aim of this retrospective population-based study was to use data extracted from the longitudinal National Health Insurance Research Database in Taiwan to compare long-term mortality between patients on PMV with and without tracheostomy and their related medical expenditures.

## 2. Materials and Methods

### 2.1. Data Source and Study Sample

This retrospective population-based study employed data extracted from 1 million randomly sampled beneficiaries recorded in the NHIRD from 2001 to 2013. The NHIRD, maintained by the National Health Research Institute of Taiwan, contains all original medical claims of all enrollees in the universal NHI program. The diagnostic codes used in this database are in the form of the International Classification of Diseases, 9th Revision, Clinical Modification (ICD-9-CM). The overall survival of the patients with tracheostomy was assessed over a 5-year observation period. Information regarding newly developed respiratory failure of ventilator support patients based on the ICD-9-CM codes (96.70, period of ventilation unspecified; 96.71, ventilated for <96 h; 96.72, ventilated for >96 h) was extracted from the NHIRD from 1 January 2002 to 31 December 2008. In total, 10,705 patients were included in the study, of which 1372 patients underwent tracheostomy (n = 563) or translaryngeal intubation (n = 779) within the study period.

### 2.2. Variables

Basic demographic data, including age, sex, socioeconomic status, premium-based monthly salary, and insured occupation, were collected. Urbanization level was measured by using a 7-point scale, with levels 1 and 7 indicating the highest and lowest urbanization levels, respectively. The health status, including pneumonia, COPD, congestive heart failure (CHF), chronic renal disease, neurovascular disease, and neuromuscular disease, was noted.

### 2.3. Outcome Measurement

The outcome measurements included mortality and medical costs. Mortality was dichotomous and categorically defined in the study.

### 2.4. Statistical Analysis

Descriptive analysis was performed to calculate the mean, standard difference frequency, percentage, and variable distribution. The chi-square test was used to compare categorical variables. Logistic regression was used to estimate the odds ratios and 95% confidence intervals (CIs) for successful weaning from a ventilator or tracheostomy. The Cox proportional hazard model was used to compare survival in the patients with tracheostomy and translaryngeal intubation after adjusting for potential confounders. The general linear model was used to calculate the difference in medical expenditures between patients with tracheostomy and translaryngeal intubation. All tests were performed in the SAS statistical software package (version 9.4; SAS Institute Inc., Cary, NC, USA), and *p*-values of <0.05 were taken to be indicative of statistical significance.

## 3. Results

**Figure 1:**
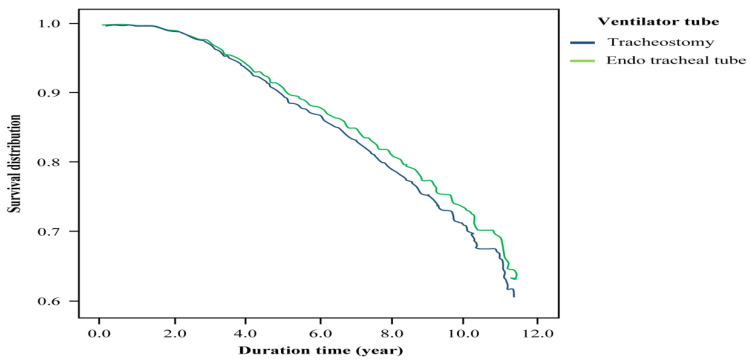
Survival curve of patients with PMV.

Altogether, we examined 1342 patients, among which 563 underwent tracheostomy, with 142 deaths, and 779 underwent translaryngeal intubation (using endotracheal tube), with 204 deaths. The total death toll was 346 (25.78%). The average survival was 4.98 years for patients with tracheostomy and 5.48 years for patients with translaryngeal intubation (Table 2), with no difference in mortality between the groups (Table 3). Sex, age, premium-based monthly salary difference, occupation, urbanization levels, COPD, CHF, chronic renal disease (CRD), and cerebrovascular diseases were statistically significant in the mortality of patients with endotracheal tubes (Table 3). In the Cox regression model, male sex (hazard ratio (HR): 1.29, 95% CI: 1.03–1.63), CHF (HR: 1.29, 95% CI: 1.02–1.63), and CRD (HR: 1.54, 95% CI: 1.10–2.14) were associated with higher mortality. Age >45 years was associated with higher mortality than age ≤45 years. Urbanization levels lower than the highest level were associated with lower mortality (Table 4). Although the total medical expenditure was higher for patients with tracheostomy than for laryngeal patients with endotracheal tubes, this was not the case for annual medical expenditures (Table 5). In the Kaplan–Meier survival curve, as shown in Figure 1 for the corrected survival curve of people using different breathing circuits, the observation time was >4 years, and patients with endotracheal tubes had a higher mortality risk than those with tracheal tubes.

## 4. Discussion

As found in previous studies, old age was a risk factor for mortality [22,23,24,25], with 40% mortality in patients >75 years old. Additionally, the mortality rate was significantly higher in lower-income patients. According to the definition of the attachment population of the Ministry of Justice and Health Insurance Bureau in Taiwan, the attachment population comprises non-occupational adults or spouses, most of whom are >65 years old or <20 years old. Higher socioeconomic patients may have more resources to promote health and a decreased risk of exposure to disease-related risk factors. However, the attachment population does not accurately reflect the family economic status. Hence, this existing bias failed to explain whether the attachment population mortality rate was lower than the low-income population rate.

In our study, there were no differences between ventilator-dependent patients undergoing tracheostomy and those with a laryngeal endotracheal tube. However, age >45 years and male sex were associated with higher mortality. Old age is an established risk factor for the long-term outcome in intensive care unit survival and patients with PMV [26,27]. In Taiwan, males tend to have higher rates of mortality, smoking, and drinking, as well as a higher body mass index than females [28,29]. Due to this unhealthy lifestyle, perhaps it is not surprising that males have a higher mortality rate.

Furthermore, CHF and CRD in patients with PMV were associated with a higher mortality rate. Patients with COPD were not only difficult to wean from the ventilator [14,30] but COPD was also recognized as an independent risk factor for the long-term mortality of ventilator-dependent patients [31,32]. In the literature, COPD and pneumonia were two common reasons for ventilator dependency [14,15] in patients with high-risk pneumonia having short- and long-term mortality [33,34]. COPD is an important risk factor accounting for the long-term mortality of patients with pneumonia in the literature [35,36,37]. However, COPD and pneumonia were not found to be significantly associated with higher mortality in our study. COPD in this study was defined according to the ICD-9-CM codes rather than spirometry or medication results [38], so there was a risk of misclassification, which is a limitation of this study.

In a population-based study, acute kidney injury with dialysis increased the long-term mortality of patients with PMV [39], whereas another population-based study showed that end-stage renal disease did not increase the long-term mortality of patients who had been ventilated, but this study did not consider patients with PMV [40]. A cohort study by Lai et al. showed that hemodialysis was associated with higher mortality in patients with PMV [25], similar to the present study findings. Few studies have assessed the relationship between CHF and the long-term outcome of patients with PMV. A retrospective study in Taiwan showed that patients with PMV and heart failure had a lower weaning rate and a higher mortality rate [11]. However, more large-scale prospective studies are required to confirm the role of CHF in patients with PMV.

The benefits of the timing of tracheostomy remain controversial. Early tracheostomy may shorten the duration of ventilator usage [17,41,42,43] but has not been found to influence mortality [17]. Young et al. reported that the early tracheostomy group did not have a decreased 30-day mortality rate [18], and Siempos et al. found that there was no difference in the 1-year mortality rates between patients with and without tracheostomy [19]. Previous studies have focused on short-term mortality or 1-year mortality, whereas the present study assessed 5-year mortality and found no difference in the long-term mortality rates between patients with translaryngeal intubation and tracheostomy, indicating that the tracheostomy procedure is safe and does not influence long-term mortality.

Patients with PMV medical costs accounted for 20.6% of the medical expenditures of the top-ten chronic illnesses requiring hospitalization in Taiwan [44]. The mean survival time was 4.98 years after tracheostomy and 5.48 years for patients with translaryngeal intubation. Additionally, there was no significant difference in the annual medical expenditures between patients with PMV and tracheostomy and translaryngeal intubation. In addition to the timing of tracheostomy, these results provide scientific evidence that physicians, patients, families, and authorities can review to assess the value of undergoing a tracheostomy in patients with PMV.

In the present study, there was no significant difference in the total cost or annual medical cost of patients with COPD, whereas a previous matched cohort study found that malnutrition status had higher medical costs in non-ventilated patients with COPD [45]. We did not extract nutritional status data in the current study, so malnutrition status should be considered to be a potential risk factor for increased medical costs in ventilated patients with COPD and evaluated in a future study.

### Limitations

There were some study limitations that should be considered. First, this was a retrospective databank study, with little information collected prospectively. Second, all diagnosis and comorbidity data were retrieved from the ICD-9 code, so misclassification or diagnosis due to reimbursement purposes in some hospitals was possible.

## 5. Conclusions

This study showed that male sex, old age, and comorbidities, including CHF and CRD, were risk factors associated with higher mortality in patients with PMV. The decision to perform a tracheostomy did not influence long-term survival or annual medical costs of patients with PMV, emphasizing that individual treatment plans should be designed to reduce mortality and medical expenditures.

## Figures and Tables

**Table 1:** Comparison of patient characteristics between tracheostomy and translaryngeal intubation.

	Tracheostomy	Translaryngeal Intubation
Style	Neck operation wound	Oral route
Quality of life	Improved oral comfort Maintained ability to eat and talk by mouth after training	Inability to close the mouth Difficulty in oral cleaning Damaged facial skin Inability to speak and communicate
Complications	Hemorrhage Loss of airway Wound infection	Oral ulcer Granuloma in the throat Damaged vocal cords

**Table 2:** Descriptive analysis of survival in patients with tracheostomy and translaryngeal intubation (*n* = 1342).

Variables	Total (n, %)	Mortality (n, %)	Average Survival (Year ± SD)
PMV	1342 (100%)	346 (25.78%)	5.27 ± 2.36
Translaryngeal intubation	779 (58.95%)	204 (26.19%)	5.48 ± 2.42
Tracheostomy	563 (41.04%)	142 (25.22%)	4.98 ± 2.25

**Table 3:** Univariate analysis of patients with PMV characteristics.

Variables	Total (N)	Survived		Mortality		χ^2^	*p*-Value
		(n)	(%)	(n)	(%)		
All	1342	996	74.22	346	25.78		
Patients with PMV						0.159	0.690
Translaryngeal intubation	779	575	73.81	204	26.19		
Tracheostomy	563	421	74.78	142	25.22		
Sex						10.102	<0.01
Female	527	416	78.94	111	21.06		
Male	815	580	71.17	235	28.83		
Age (years)						92.081	<0.001
<45	285	260	91.23	25	8.77		
45–54	160	130	81.25	30	18.75		
55–64	167	128	76.65	39	23.35		
65–74	327	237	72.48	90	27.52		
75–84	324	193	59.57	131	40.43		
≥85	79	48	60.76	31	39.24		
Premium-based monthly salary (NTD)						51.228	<0.001
≤20,008	436	275	63.07	161	36.93		
Dependent population	651	500	76.8	151	23.2		
≥20,009	255	221	86.67	34	13.33		
Occupation						26.362	<0.001
First category (private employee and government)	53	46	86.79	7	13.21		
Second category (labor union member)	56	51	91.07	5	8.93		
Third category (farmer and fisherman)	169	141	83.43	28	16.57		
Fourth and fifth categories (soldier and social)	89	59	66.29	30	33.71		
Sixth category (veteran and religious group)	975	699	71.69	276	28.31		
Urbanization level						30.1064	<0.001
Level 1	320	213	66.56	107	33.44		
Level 2	389	274	70.44	115	29.56		
Level 3	248	190	76.61	58	23.39		
Level 4	202	167	82.67	35	17.33		
Level 5	41	37	90.24	4	9.76		
Level 6	85	70	82.35	15	17.65		
Level 7	57	45	78.95	12	21.05		
Pneumonia						0.824	0.3641
No	641	483	75.35	158	24.65		
Yes	701	513	73.18	188	26.82		
COPD						18.225	<0.001
No	864	674	78.01	190	21.99		
Yes	478	322	67.36	156	32.64		
Congestive heart failure						26.520	<0.001
No	823	651	79.1	172	20.9		
Yes	519	345	66.47	174	33.53		
Chronic renal disease						10.235	<0.01
No	1,227	925	75.39	302	24.61		
Yes	115	71	61.74	44	38.26		
Cerebrovascular disease						10.154	<0.01
No	803	621	77.33	182	22.67		
Yes	539	375	69.57	164	30.43		
Neuromuscular disease						0.332	0.5644
No	1,265	941	74.39	324	25.61		
Yes	77	55	71.43	22	28.57		

**Table 4:** Cox regression model of survival analysis in patients with PMV.

Variables	Unadjusted HR	95% CI			*p*-Value	Adjusted HR	95% CI			*p*-Value
Patients with PMV										
Translaryngeal intubation	1					1				
Tracheostomy	0.97	0.78	–	1.20	0.749	0.90	0.72	–	1.13	0.380
Sex										
Female (Reference)	1					1				
Male	1.36	1.08	–	1.70	<0.05	1.29	1.03	–	1.63	<0.05
Age										
<45 y/o (Reference)	1					1				
45–54 y/o	2.32	1.36	–	3.94	<0.01	2.26	1.32	–	3.88	<0.01
55–64 y/o	2.87	1.74	–	4.74	<0.001	2.76	1.64	–	4.64	<0.001
65–74 y/o	3.55	2.28	–	5.54	<0.001	3.15	1.97	–	5.05	<0.001
75–84 y/o	6.55	4.26	–	10.06	<0.001	5.03	3.13	–	8.08	<0.001
≥85 y/o	7.29	4.29	–	12.37	<0.001	6.27	3.54	–	11.11	<0.001
										
Premium-based monthly salary (NTD)										
≤20,008 Dollars (Reference)	1					1				
Dependent population	0.56	0.45	–	0.70	<0.001	0.70	0.55	–	0.90	<0.01
≥20,009	0.33	0.23	–	0.48	<0.001	0.21	0.05	–	0.81	<0.05
Occupation										
First category (private employee and government) (Reference)	1					1				
Second category (labor union member)	0.65	0.21	–	2.06	0.467	1.11	0.23	–	5.25	0.896
Third category (farmer and fisherman)	1.26	0.55	–	2.88	0.586	1.86	0.49	–	6.98	0.359
Fourth and fifth categories (soldier and social)	2.47	1.09	–	5.63	<0.05	1.02	0.42	–	2.48	0.973
Sixth categories (veteran and religious groups)	2.18	1.03	–	4.61	<0.05	0.72	0.31	–	1.68	0.450
Urbanization level										
Level 1 (Reference)	1					1				
Level 2	0.88	0.68	–	1.14	0.335	0.75	0.57	–	0.98	<0.05
Level 3	0.66	0.48	–	0.91	<0.05	0.56	0.41	–	0.78	<0.001
Level 4	0.46	0.32	–	0.68	<0.001	0.42	0.28	–	0.63	<0.001
Level 5	0.28	0.10	–	0.75	<0.05	0.25	0.09	–	0.68	<0.01
Level 6	0.50	0.29	–	0.85	<0.05	0.40	0.23	–	0.71	<0.01
Level 7	0.62	0.34	–	1.13	0.120	0.49	0.27	–	0.92	<0.05
Pneumonia										
No (Reference)	1					1				
Yes	1.29	1.04	–	1.59	<0.05	1.02	0.81	–	1.28	0.898
COPD										
No (Reference)	1					1				
Yes	1.86	1.50	–	2.30	<0.0001	1.27	1.00	–	1.61	0.052
Congestive heart failure										
No (Reference)	1					1				
Yes	2.02	1.63	–	2.49	<0.0001	1.29	1.02	–	1.63	<0.05
Chronic renal disease										
No (Reference)	1					1				
Yes	1.95	1.42	–	2.68	<0.001	1.54	1.10	–	2.14	<0.05
Cerebrovascular disease										
No (Reference)	1					1				
Yes	1.57	1.27	–	1.94	<0.001	1.19	0.95	–	1.49	0.133
Neuromuscular disease										
No (Reference)	1					1				
Yes	1.20	0.78	–	1.85	0.404	1.21	0.78	–	1.88	0.393

**Table 5:** Linear regression model of medical costs in patients with PMV.

Variables	Total Medical Cost					Annual Medical Cost				
	Unadjusted	*p*-Value	Adjusted		*p*-Value	Unadjusted	*p*-Value	Adjusted		*p*-Value
	Coefficients		Average Value of Smallest Square	Coefficients		Coefficients		Average Value of Smallest Square	Coefficients	
Patients with PMV										
Translaryngeal intubation (Reference)			1,663,197.09					2,177,477.92		
Tracheostomy	1,215,160	<0.001	2,759,195.31	1,095,998	<0.001	−1581	0.995	2,129,391.83	−48,086	0.839
Sex										
Female (Reference)			2,116,280.54					2,301,919.93		
Male	215,556	0.189	2,306,111.86	189,831	0.241	−266,942	0.250	2,004,949.82	−296,970	0.200
Urbanization level										
Level 1 (Reference)			2,397,712.55					1,729,964.34		
Level 2	47,102	0.911	2,642,481.77	−48,459	0.908	2,640,012	<0.001	1,773,750.38	2,680,525	<0.001
Level 3	−162,251	0.649	2,238,771.07	−122,630	0.731	−71,043	0.888	1,807,407.71	94,273	0.854
Level 4	−908,669	0.061	1,917,074.72	−739,716	0.126	−167,666	0.806	1,787,265.82	10,965	0.987
Level 5	−584,933	<0.05	1,657,996.87	−480,638	0.070	−115,100	0.756	1,740,928.95	57,301	0.880
Level 6	−119,316	0.630	2,275,082.95	−158,941	0.516	−13,395	0.969	1,824,237.79	77,443	0.825
Level 7	299,320	0.175	2,349,253.48	244,769	0.257	59,010	0.850	4,410,489.13	43,786	0.887
Pneumonia										
No (Reference)			2,047,683.86					2,238,768.41		
Yes	516,672	<0.01	2,374,708.53	327,025	<0.05	−141,574	0.533	2,068,101.34	−170,667	0.469
COPD										
No (Reference)			2,143,378.14					2,146,556.23		
Yes	235,296	0.160	2,279,014.26	135,636	0.458	−89,303	0.706	2,160,313.52	13,757	0.958
Congestive heart failure										
No (Reference)			1,952,943.31					2,118,083.55		
Yes	392,577	<0.05	2,469,449.09	516,506	<0.01	−72,350	0.756	2,188,786.19	70,703	0.786
Chronic renal disease										
No (Reference)			2,193,369.16					2,134,248.28		
Yes	39,173	0.891	2,229,023.24	35,654	0.901	−45,400	0.911	2,172,621.47	38,373	0.925
Cerebrovascular disease										
No (Reference)			2,214,315.27					2,186,823.15		
Yes	219,130	0.180	2,208,077.13	−6238	0.971	−125,437	0.587	2,120,046.59	−66,777	0.786
Neuromuscular disease										
No (Reference)			1,957,674.33					2,125,552.21		
Yes	789,420	<0.05	2,464,718.07	507,044	0.135	25,825	0.958	2,181,317.54	55,765	0.909

## Data Availability

The datasets used in this study are available from the Ministry of Health and Welfare, Taiwan, on reasonable request.

## References

[1] Ranes J.L., Gordon S.M., Chen P., Fatica C., Hammel J., Gonzales J.P., Arroliga A.C. (2006). Predictors of long-term mortality in patients with ventilator-associated pneumonia. Am. J. Med..

[2] Holzapfel L., Chevret S., Madinier G., Ohen F., Demingeon G., Coupry A., Chaudet M. (1993). Influence of long-term oro- or nasotracheal intubation on nosocomial maxillary sinusitis and pneumonia: Results of a prospective, randomized, clinical trial. Crit. Care Med..

[3] Cavaliere S., Bezzi M., Toninelli C., Foccoli P. (2007). Management of post-intubation tracheal stenoses using the endoscopic approach. Monaldi Arch. Chest Dis..

[4] Heffner J.E., Hess D. (2001). Tracheostomy management in the chronically ventilated patient. Clin. Chest Med..

[5] Plummer A.L., Gracey D.R. (1989). Consensus conference on artificial airways in patients receiving mechanical ventilation. Chest.

[6] Freeman-Sanderson A.L., Togher L., Elkins M.R., Phipps P.R. (2016). Return of voice for ventilated tracheostomy patients in ICU: A randomized controlled trial of early-targeted intervention. Crit. Care Med..

[7] Freeman B.D. (2017). Tracheostomy update: When and how. Crit. Care Clin..

[8] Terragni P.P., Antonelli M., Fumagalli R., Faggiano C., Berardino M., Pallavicini F.B., Miletto A., Mangione S., Sinardi A.U., Pastorelli M. (2010). Early vs. late tracheotomy for prevention of pneumonia in mechanically ventilated adult ICU patients: A randomized controlled trial. JAMA.

[9] Scales D.C., Ferguson N.D. (2010). Early vs. late tracheotomy in ICU patients. JAMA.

[10] MacIntyre N.R., Epstein S.K., Carson S., Scheinhorn D., Christopher K., Muldoon S. (2005). National Association for Medical Direction of Respiratory Management of patients requiring prolonged mechanical ventilation: Report of a NAMDRC consensus conference. Chest.

[11] Wu Y.K., Kao K.C., Hsu K.H., Hsieh M.J., Tsai Y.H. (2009). Predictors of successful weaning from prolonged mechanical ventilation in Taiwan. Respir. Med..

[12] Wunsch H., Linde-Zwirble W.T., Angus D.C., Hartman M.E., Milbrandt E.B., Kahn J.M. (2010). The epidemiology of mechanical ventilation use in the United States. Crit. Care Med..

[13] Schönhofer B., Euteneuer S., Nava S., Suchi S., Köhler D. (2002). Survival of mechanically ventilated patients admitted to a specialised weaning centre. Intensive Care Med..

[14] Thille A.W., Boissier F., Ghezala H.B., Razazi K., Mekontso-Dessap A., Brun-Buisson C. (2014). Risk factors for and prediction by caregivers of extubation failure in ICU patients: A prospective study. Crit. Care Med..

[15] McConville J.F., Kress J.P. (2012). Weaning patients from the ventilator. N. Engl. J. Med..

[16] Namen A.M., Ely E.W., Tatter S.B., Case L.D., Lucia M.A., Smith A., Landry S., Wilson J.A., Glazier S.S., Branch C.L. (2001). Predictors of successful extubation in neurosurgical patients. Am. J. Respir. Crit. Care Med..

[17] Koch T., Hecker B., Hecker A., Brenck F., Preuß M., Schmelzer T., Padberg W., Weigand M.A., Klasen J. (2012). Early tracheostomy decreases ventilation time but has no impact on mortality of intensive care patients: A randomized study. Langenbecks Arch. Surg..

[18] Young D., Harrison D.A., Cuthbertson B.H., Rowan K. (2013). Effect of early vs. late tracheostomy placement on survival in patients receiving mechanical ventilation: The TracMan randomized trial. JAMA.

[19] Siempos I.I., Ntaidou T.K., Filippidis F.T., Choi A.M.K. (2015). Effect of early versus late or no tracheostomy on mortality and pneumonia of critically ill patients receiving mechanical ventilation: A systematic review and meta-analysis. Lancet Respir. Med..

[20] Pilcher D.V., Bailey M.J., Treacher D.F., Hamid S., Williams A.J., Davidson A.C. (2005). Outcomes, cost and long term survival of patients referred to a regional weaning centre. Thorax.

[21] Mehta A.B., Syeda S.N., Bajpayee L., Cooke C.R., Walkey A.J., Wiener R.S. (2015). Trends in tracheostomy for mechanically ventilated patients in the United States, 1993–2012. Am. J. Respir. Crit. Care Med..

[22] Engoren M.C., Arslanian-Engoren C.M. (2005). Outcome after tracheostomy for respiratory failure in the elderly. J. Intensive Care Med..

[23] Spicher J.E., White D.P. (1987). Outcome and function following prolonged mechanical ventilation. Arch. Intern. Med..

[24] Carson S.S., Garrett J., Hanson L.C., Lanier J., Govert J., Brake M.C., Landucci D.L., Cox C.E., Carey T.S. (2008). A prognostic model for one-year mortality in patients requiring prolonged mechanical ventilation. Crit. Care Med..

[25] Lai C.C., Shieh J.M., Chiang S.R., Chiang K.H., Weng S.F., Ho C.H., Tseng K.L., Cheng K.C. (2016). The outcomes and prognostic factors of patients requiring prolonged mechanical ventilation. Sci. Rep..

[26] Chelluri L., Im K.A., Belle S.H., Schulz R., Rotondi A.J., Donahoe M.P., Sirio C.A., Mendelsohn A.B., Pinsky M.R. (2004). Long-term mortality and quality of life after prolonged mechanical ventilation. Crit. Care Med..

[27] Herridge M.S., Chu L.M., Matte A., Tomlinson G., Chan L., Thomas C., Friedrich J.O., Mehta S., Lamontagne F., Levasseur M. (2016). The RECOVER program: Disability risk groups and 1-year outcome after 7 or more days of mechanical ventilation. Am. J. Respir. Crit. Care Med..

[28] National Health Service, Ministry of Health and Welfare, R.O.C. (Taiwan) 2017 Health and Welfare Gender Statistic Tables and Figures. https://dep.mohw.gov.tw/dos/lp-1723-113-1-20.html.

[29] National Health Service, Ministry of Health and Welfare, R.O.C. (Taiwan) 2016 Annual Report. https://www.mohw.gov.tw/lp-137-2.html.

[30] Boles J.M., Bion J., Connors A., Herridge M., Marsh B., Melot C., Pearl R., Silverman H., Stanchina M., Vieillard-Baron A. (2007). Weaning from mechanical ventilation. Eur. Respir. J..

[31] Marchese S., Lo Coco D., Lo Coco A. (2008). Outcome and attitudes toward home tracheostomy ventilation of consecutive patients: A 10-year experience. Respir. Med..

[32] Kojicic M., Li G., Ahmed A., Thakur L., Trillo-Alvarez C., Cartin-Ceba R., Gay P.C., Gajic O. (2011). Long-term survival in patients with tracheostomy and prolonged mechanical ventilation in Olmsted County, Minnesota. Respir. Care.

[33] Restrepo M.I., Faverio P., Anzueto A. (2013). Long-term prognosis in community-acquired pneumonia. Curr. Opin. Infect. Dis..

[34] Grijalva C.G. (2015). Is pneumonia a risk factor or a risk marker for long-term mortality?. Am. J. Respir. Crit. Care Med..

[35] Holter J.C., Ueland T., Jenum P.A., Müller F., Brunborg C., Frøland S.S., Aukrust P., Husebye E., Heggelund L. (2016). Risk factors for long-term mortality after hospitalization for community-acquired pneumonia: A 5-year prospective follow-up study. PLoS ONE.

[36] Guertler C., Wirz B., Christ-Crain M., Zimmerli W., Mueller B., Schuetz P. (2011). Inflammatory responses predict long-term mortality risk in community-acquired pneumonia. Eur. Respir. J..

[37] Kózka M., Sega A., Wojnar-Gruszka K., Tarnawska A., Gniadek A. (2020). Risk factors of pneumonia associated with mechanical ventilation. Int. J. Environ. Res. Public Health.

[38] Shantakumar S., Pwu R.F., D’Silva L., Wurst K., Kuo Y.W., Yang Y.Y., Juan Y.C., Chan K.A. (2018). Burden of asthma and COPD overlap (ACO) in Taiwan: A nationwide population-based study. BMC Pulm. Med..

[39] Chao C.T., Hou C.C., Wu V.C., Lu H.M., Wang C.Y., Chen L., Kao T.W. (2012). The impact of dialysis-requiring acute kidney injury on long-term prognosis of patients requiring prolonged mechanical ventilation: Nationwide population-based study. PLoS ONE.

[40] Chen C.M., Lai C.C., Cheng K.C., Weng S.F., Liu W.L., Shen H.N. (2015). Effect of end-stage renal disease on long-term survival after a first-ever mechanical ventilation: A population-based study. Crit. Care.

[41] Griffiths J., Barber V.S., Morgan L., Young J.D. (2005). Systematic review and meta-analysis of studies of the timing of tracheostomy in adult patients undergoing artificial ventilation. BMJ.

[42] Avilés-Jurado F.X., Prieto-Alhambra D., González-Sánchez N., de Ossó J., Arancibia C., Rojas-Lechuga M.J., Ruiz-Sevilla L., Remacha J., Sánchez I., Lehrer-Coriat E. (2021). Timing, complications, and safety of tracheotomy in critically ill patients with COVID-19. JAMA Otolaryngol. Head Neck Surg..

[43] Wang R., Pan C., Wang X., Xu F., Jiang S., Li M. (2019). The impact of tracheotomy timing in critically ill patients undergoing mechanical ventilation: A meta-analysis of randomized controlled clinical trials with trial sequential analysis. Heart Lung.

[44] National Health Insurance Administration R.O.C. (2016). 2015 Health Insurance Data. http://www.nhi.gov.tw/webdata/webdata.aspx?menu=17&menu_id=1023&WD_ID=1043&webdata_id=5365.

[45] Jerng J.S., Tang C.H., Cheng R.W., Wang M.Y., Hung K.Y. (2019). Healthcare utilization, medical costs and mortality associated with malnutrition in patients with chronic obstructive pulmonary disease: A matched cohort study. Curr. Med. Res. Opin..

